# Chloroform—Its Action and Administration

**Published:** 1866-02

**Authors:** 


### CHLOROFORM—ITS ACTION AND ADMINISTRATION.

By Arthur Earnest Sansom, M. B. Lond. Philadelphia: Lindsay & Blakiston. 1866. Pp. xii., 279.

Of the twenty chapters of this book, seven discuss the dangers of chloroform, the nature and mode of death from its use, and the methods of resuscitation therefrom. This ought to be its all-sufficient commentary.

One of the warmest and most polemical advocates of chloroform, Mr. Charles Kidd, in September, 1865, conceded 250 deaths to have been caused by chloroform.* Mr. San-

*The Philadelphia *Medical and Surgical Reporter*, January 13th, 1866, quotes Mr. Kidd, as saying, "that during this year (1865) he had extractsom, remarking that it has been impossible to obtain the records of all the fatal cases, admits but 109, that being the number, the facts of which have been collected by the recent Chloroform Committee of the Royal Medico-Chirurgical Society. In these death occurred

| | |
|---|---|
| Before the full effect of chloroform was obtained, in - - | 50 |
| During the effect, in - - - - - - - - - - | 52 |
| At a period not stated, in - - - - - - - - | 7 |
| | 109 |

In 72 instances the victims were males and in 37 females. In 62 cases, the chloroform was administered for "minor causes."

In explanation of these striking statements, the author says: "Nature provides in disease a gradual depression of the functions; an accommodation by degrees to a less perfect life. An animal which has been gradually accustomed to breathe an impure atmosphere will continue alive, though another healthy one plunged therein will die immediately." We fail to perceive the force of this theory, but Mr. Sansom argues that hence "the feeble bear chloroform better than the strong; children are the best subjects of all; women are better than men; men are most prone to death when they are in the prime of life; and when they are debilitated by pre-existing disease their chance is better than when they submit to chloroform for trivial causes in more perfect health and strength; and, further, the chance of ultimate recovery is greater when an operation is done under chloroform for a diseased condition than when it is undertaken for an injury."

The indiscriminate administration of chloroform is asserted to be dangerous; the intemperate, those having fatty hearts, the nervous and hysterical, and those suffering under shock of the nervous system, are pronounced, by Mr. Sansom, to be especially prone to suffer the accidents of chloroform administration.

"Experiments upon the lower animals, equally with observations on man, prove that there is but a narrow limit between that strength in which the vapor may be safely inhaled and that which is likely to produce alarming symped notes from various newspapers and medical journals of over a dozen deaths by chloroform, 'deaths lying *perdu*, as it were, or hidden, but all bearing out the views of 250 others that he had in vain collected and offered the British medical and other weeklies.'"

toms, if not death." Consequently, nothing but the most approved inhaler should ever be used, and an atmosphere, containing four and a half per cent. of chloroform, is all that can, with safety, be employed; this ratio should be sustained by mechanical means and by no guess-work contrivances." In thirty-six deaths, the average amount of chloroform used was 1.7 drachms."

We have selected these paragraphs for citation to show that the book furnishes us with no new ideas of chloroform, or affords any re-assurance in its use. On the contrary, it adds to our pre-existing fears; it appals us by the dangers it describes; it intimidates by the extreme caution demanded in its exhibtion, by the failure of the greatest care to avert a fatal result.

The compromise adopted by the Chloroform Committee, above alluded to, accepted and approved by Mr. Sansom, of a mixture of sulphuric ether and chloroform, is, we conceive, if not a triumph on the part of ether, at least a tacit condemnation of chloroform. That Committee remark that "it is quite possible that at some future time an anæsthetic may be discovered which will fulfil the required condition yet more perfectly than these mixtures." That discovery was made on the 13th of September, 1846 (at 19 Tennant's Row, Boston, Mr. Sansom has it), and has been in daily use since November 7th, of the same year, when the first capital operation, in which pain was totally annulled, was performed at the Massachusetts General Hospital, one year, less three days, before the anæsthetic properties of chloroform were discovered. From that day to this not a single unavoidable accident has resulted in its use, nor has it ever failed, in hands familiar with its administration, to produce complete, satisfactory and perfectly safe anæsthesia. The converse of all which relates to the danger of chloroform is true of ether. Where chloroform threatens, sulphuric ether affords entire immunity from danger, and where chloroform is safe, sulphuric ether is safer; and if, as stated by Mr. Sansom, six minutes is the average time required to accomplish anæsthesia by chloroform, one of the chief arguments hitherto urged in its favor, as compared with sulphuric ether, is destroyed, since that is a liberal allowance of time to easily effect a thorough etherization.

In this connection, and as bearing on the question of the practical use of ether, the recently published statistics, contained in "Circular No, 6, War Department, Surgeon General's office, Nov. 1, 1865, (p. 87), are interesting. It appears that the reports of 23,260 surgical operations have been consulted in regard to the employment of anæsthetics. "Chloroform was used in 60 per cent. of these, ether in 30 per cent., and in 10 per cent., a mixture of the two was administered (in 40 per cent., a more bulky anæsthetic than chloroform was adopted). At the general hospitals, the greater safety of ether as an anæsthetic was commonly conceded. It was often employed, and no fatal accident from its use has been reported. In the field operations chloroform was almost exclusively used. In seven instances fatal results have been ascribed with apparent fairness to its use. Detailed reports of these unfortunate cases are on file."

We have only to say, in conclusion, that Mr. Sansom appears to have written a complete and candid treatise on the subject of chloroform. We regret to think that a firm, usually so judicious, should have deemed it for their interest to reprint the book, or that there are, in the United States, enough persons so blind to their own interests and the safety of their patients, as to purchase even a small edition. Would the public, by purchasing, endorse the propriety of publishing an essay on the "domestic uses of gunpowder," or on "camphene and its admirable adaptation for kindling fires?" The book is well printed and on good paper, but it should have been bound in black, with a "death's head" stamped on the covers, as apothecaries label their laudanum when they dispense it in phials.—*Boston Med. and Surg. Jour.*

